# Nanoparticles and Plant By-Products for Edible Coatings Production: A Case Study with Zinc, Titanium, and Silver

**DOI:** 10.3390/polym14142837

**Published:** 2022-07-12

**Authors:** Alexandra Tauferová, Zdeňka Javůrková, Matej Pospiech, Hana Koudelková Mikulášková, Karolína Těšíková, Dani Dordevic, Simona Dordevic, Bohuslava Tremlová

**Affiliations:** Department of Plant Origin Food Sciences, Faculty of Veterinary Hygiene and Ecology, University of Veterinary Sciences Brno, Palackého tř. 1946/1, 612 42 Brno, Czech Republic; tauferovaa@vfu.cz (A.T.); javurkovaz@vfu.cz (Z.J.); pospiechm@vfu.cz (M.P.); koudelkovamih@vfu.cz (H.K.M.); tesikovak@vfu.cz (K.T.); dordevicd@vfu.cz (D.D.); dordevics@vfu.cz (S.D.)

**Keywords:** packaging, nanoparticles, sensory properties, scanning electron microscopy, CIE*Lab*, plant extract

## Abstract

For the development of functional edible packaging that will not lead to rejection by the consumer, it is needed to analyze the interactions between ingredients in the packaging matrix. The aim of this study was to develop edible chitosan-based coatings that have been enriched with red grape extracts, zinc, silver, and titanium nanoparticles. The organoleptic properties of the produced edible packaging were described by quantitative descriptive analysis and consumer acceptability was verified by hedonic analysis. By image analysis, color parameters in the CIE*Lab* system, opacity, Whiteness and Yellowness Index were described. The microstructure was described by scanning electron microscopy. The hedonic evaluation revealed that the addition of nanometals and their increasing concentration caused a deterioration in sample acceptability. The overall evaluation was higher than 5 in 50% of the samples containing nanometals. The addition of nanometals also caused statistically significant changes in L*, a*, and b* values. The sample transparency generally decreased with the increasing concentration of nanoparticle addition. Scanning electron microscopy showed, that the addition of nanometals does not disrupt the protective function of the packaging. From a sensory point of view, the addition of ZnO nanoparticles in concentrations of 0.05 and 0.2% appeared to be the most favorable of all nanometals.

## 1. Introduction

With the increasing demands on packaging technologies, the development of new types of packaging materials is becoming more important. This development raises a number of issues related to environmental pollution and waste recycling [1]. 

Therefore, attention is currently being paid to research into biodegradable packaging materials made from renewable raw materials that are environmentally friendly. Edible packaging seems to be a suitable alternative to plastic materials and represents great potential in a number of different areas [2]. 

An important factor in the development of edible packaging is the combination of knowledge of biodegradable materials and the food packaging sector. Based on the results of a number of studies, it is possible to state a significant effect of edible packaging on maintaining quality and prolonging shelf life, for example in fresh or sliced food [3]. Studies also point to the functionality of edible packaging as an effective barrier on the surface of food, which has the ability to reduce water loss or modify the internal atmosphere, which can slow down some of the processes that lead to their aging [4]. Another potential benefit of edible packaging is their use as carriers of active ingredients that increase antioxidant capacity or improve antibacterial properties that limit the growth of pathogens on the food surface [5,6].

Current research in the food sector has been significantly influenced by nanotechnology which also involves the development of functional edible food packaging. The use of nanoparticles in the production of edible packaging seems to be very promising. Individual studies deal with the use of artificial nanoparticles, which are added to food packaging as a functional component. Their indisputable advantage is increasing food stability, reducing the growth of bacteria, fungi, and yeasts, or, in some cases, protecting light-sensitive foods [6,7]. The incorporation of nanoparticles into edible materials has contributed to the development of novel edible materials called nanocomposites. Silver, zinc oxide, and titanium dioxide are among the most used inorganic nanoparticles [8]. Considering specifically the improved food packaging physical properties, the incorporation of silver nanoparticles may contribute to the increased oxygen and water vapor barrier properties, and the addition of zinc oxide nanoparticles may lead to improved mechanical and heat seal properties, both with reduced oxygen permeability. Moreover, zinc oxide nanoparticles have the potential to mask the intense aroma of antimicrobial essential oils and improve their activity. Photoinduced titanium oxide nanoparticles act as oxygen and ethylene scavengers [9]. Of course, the concentration of selected nanoparticles affects both the individual physical properties of the edible packaging and plays a vital role to achieve an adequate antimicrobial effect [10].

In addition to the functional and mechanical properties of edible packaging, an essential and often monitored parameter is their color and transparency, in particular, because of the overall appearance of the packaging which entails positive consumer reactions [11]. Consumer preferences are one of the basic factors determining the usability of new products and this also applies to the sensory properties of edible packaging, which significantly affect the acceptance of final products [12].

Although various additives may improve the functional or nutritional properties of edible packaging, they may also lead to a deterioration in the mechanical or sensory properties and, consequently, to rejection by the consumer. For the development of functional edible packaging with high sensory performance, further studies are needed to shed more light on the interactions between individual functional ingredients in the edible packaging matrix [13]. 

This study builds on previous studies confirming the suitability of adding red grape extract to chitosan-based edible packaging in terms of its superior antimicrobial properties and antioxidant capacity compared to other alternatives to the investigated plant by-products. [14]. The concentration of 10% was selected with regard to sensory acceptability, as a higher concentration of grape extract resulted in a more intense color of the packaging and thus lower sensory acceptability [15].

In view of the above, the aim of this study was to develop edible chitosan-based coatings that have been enriched with zinc, silver, and titanium nanoparticles. Red grape extracts have also been used as a suitable source of bioactive ingredients.

## 2. Materials and Methods

### 2.1. Packaging Preparation 

#### 2.1.1. Production of Packaging with the Addition of Nanoparticles

ZnO and TiO_2_ nanoparticles were weighed for packaging preparation in concentrations of 0.05, 0.2, and 0.5%. Subsequently, 135.0 mL of 1.0% lactic acid dissolved in distilled water was added with the addition of 1.5 g of chitosan. The mixture was then heated and stirred on a magnetic stirrer for 15 min at 50 °C at 750 rpm, and afterward, 0.75 mL of glycerol was added and stirred again for 5 min. The prepared film-forming solution was poured into a 15.0 cm diameter Petri dish, where it was allowed to dry for 48 h. The preparation procedure was similar for samples containing the red grape extract. After stirring for 15 min, 13.5 mL of the extract was added, and at the same time, 13.5 mL less lactic acid was initially used. 

#### 2.1.2. Production of Packaging with the Addition of Colloidal Silver 

To prepare edible packaging with the addition of colloidal silver, 1.5 g of chitosan was weighed and 135.0 mL of 1% lactic acid dissolved in colloidal silver at concentrations of 10, 30, and 50 ppm was added (Table 1). The mixture was stirred and then heated on a magnetic stirrer for 15 min at 50 °C and 750 rpm. Subsequently, 0.75 mL of glycerol was added, stirred for 5 min, and poured into 15.0 cm diameter Petri dishes, where they were allowed to dry for 48 h. The thickness of the experimentally produced edible packaging was 0.21 ± 0.01 mm. 

#### 2.1.3. Grape Marc Preparation 

Red grapes of Scarlotta Seedless variety grown in South Africa and purchased in the regular market network (Tesco, Brno, Czech Republic) were used for the production of marc. The juice was obtained from the red grapes using a Catler JE 4011 juicer. The by-product of the juice production, the marc, was then transferred to bags and frozen for further use.

#### 2.1.4. Extract Preparation 

From the thawed marc, 10.0 g was weighed to which 100.0 mL of boiling distilled water (100 °C) was added, and 10 min later filtration was performed (Whatmann KA-1 filter paper), the extract at room temperature was then used to prepare the packaging. 

### 2.2. Sensory Analysis

The key organoleptic properties of edible packaging were described by quantitative descriptive analysis and consumer acceptability was verified by hedonic analysis. As the actual use of edible packaging in practice to date is only small, a questionnaire survey was conducted among the evaluators in order to research deeper their perception of the use of produced edible packaging for selected food groups.

#### 2.2.1. Quantitative Descriptive Analysis

Quantitative descriptive analysis and hedonic analysis were performed at the Institute of Plant Hygiene and Food Technology, FVHE, VETUNI. A panel of 14 trained evaluators, who had previous experience in evaluating edible packaging, participated in the quantitative descriptive analysis. For the purpose of quantification of attributes, 9-degree categorical ordinal scales with described extremes from 1 (minimum intensity) to 9 (highest intensity) were used. The evaluated descriptors included color intensity, odor intensity, surface character, flexibility, stickiness, and overall rating. Quantitative descriptive analysis was performed twice.

#### 2.2.2. Hedonic Analysis

Based on the results of the analysis performed by the trained panel, 11 samples were selected from a total of 20 samples, which achieved an average score higher than 5 in the overall evaluation. These 11 selected samples were subsequently evaluated by hedonic analysis, in which 65 untrained evaluators from among students and employees of the University of Veterinary Sciences Brno participated. Descriptors including pleasantness of appearance, pleasantness of aroma, pleasantness of texture, and overall evaluation were measured using a 9-digit category ordinal hedonic scale, where 1 meant an extremely unpleasant sensation, 5 meant a neutral sensation, and 9 the highest degree of pleasantness. 

#### 2.2.3. Assessment of the Probability of Purchasing Food Commodities in Edible Packaging

The hedonic evaluation also assessed the probability of purchasing products, such as fruit, vegetables, meat products, bakery products, and milk products (cheese), in individual samples of edible packaging. For the purposes of this evaluation, a 5-point scale was used, where 1 meant the lowest willingness to consume the product in the given edible packaging, 3 meant a neutral attitude, and 5 meant that the evaluator would certainly be willing to consume the given commodity in the given packaging.

### 2.3. Evaluation of Color and Color Properties of Packaging

The samples were placed in 150.0 mm Petri dishes. Digital images of all samples were obtained by a computer vision system. Scanning was performed under standard lighting conditions, which were provided by Osram Delux L—1 × 18 W lamps (OSRAM GmbH., Munich, Germany) and scanning in a dark chamber. Individual images were taken by a Canon EOS 600D camera (Canon, Tokyo, Japan) mounted on a tripod (Fomei CS 920, Hradec Králové, Czech Republic) against a white and a black background. The shooting mode was in manual setting: exposure time 1/15, aperture F 8.0, image size L, sensitivity ISO 100 [16]. Each sample was captured 10 times. 

Subsequently, the images were processed by Nikon Imaging Software NIS-Elements BR 4.13.04 (Laboratory Imaging s.r.o., Prague, CZE). The same area of the circle (ROI—region of interest) was always selected for evaluation within NIS-Elements. Subsequently, the color characteristics of MeanRed, MeanGreen, and MeanBlue were measured and then converted to CIEL*a*b* system where L* means lightness, a* indicates the position on the red–green axis, and b* on the yellow–blue axis. 

The difference between samples and control (ΔE) was also monitored. This parameter was calculated using the equation of CIE ΔE2000 [17,18]. 

#### 2.3.1. Opacity

Another monitored parameter was the opacity of the packaging. This was obtained by calculation according to the following Equation (1):

(1) $$\%\;Opacity=\frac{\left(L*black\right)}{\left(L*white\right)}*100$$

where L* black was obtained from the measurement values of images taken on a black background and L* white on a white background. A value of 100% indicates opaque packaging and a value of 0% indicates transparent packaging [19].

#### 2.3.2. Whiteness Index

Another parameter was the Whiteness Index, which was obtained by Equation (2) given in Li et al., 2019 [20]:

(2) $$Whiteness\;Index=100-\sqrt{\;\;{\left(100-L*\right)}^{2}+a{*}^{2}+b{*}^{2}}$$

where L* is the value obtained from the measurement calculations on the white background, a* indicates the position on the red–green axis, and b* on the yellow–blue axis.

#### 2.3.3. Yellowness Index

In their work, Saberi et al. (2016) used the parameter of the Yellowness Index for the evaluation. This parameter is obtained by the following, Equation (3) [21]:

(3) $$\mathrm{Yellowness}\;\mathrm{Index}=\frac{142.86\;b*}{L*}$$

where L* is the value obtained from the measurement calculations on the white background and b* indicates the position on the yellow–blue axis.

### 2.4. Evaluation of the Surface of Edible Packaging by SEM

The microstructure of experimentally produced edible packaging was described by SEM, primarily the particle distribution, crystal formation, degree of phase segregation, and the formation of cracks related to the barrier properties of the packaging.

The surface and fracture of the edible packaging were evaluated. The surface evaluation of the edible packaging was performed after forming the gel on a conductive target so as to minimize surface changes caused by bending or fracturing during handling. 

For fracture analysis, the edible film formed in the standard way (in compliance with Section 2.1.1 and Section 2.1.2) was frozen with liquid nitrogen and subsequently mechanically broken. The fragments were applied to a carbon double-sided adhesive tape. The samples were gilded with 10 nm on a Q150R ES sample plating device (Quorum Technologies, Laughton, UK) and subsequently examined in triplicate with a MIRA3 TESCAN microscope (Tescan, a.s., Brno, Czechoslovakia), at a voltage of 5.0 kV. Magnification 1kx, 10kx and refraction, 3kx surface images. The exact magnification is shown on the individual micrographs.

Total area and nearest fissure distance were measured on microphotographs by the imaging analysis software NIS Element (Laboratory Imagine, Prague, Czechoslovakia). The nearest object’s distance was calculated as the smallest distance to another object (measured between their centers of gravity). The area was calculated by the following, Equation (4):

(4) Area=∑pixel

### 2.5. Statistical Analysis

The results of the sensory analysis of the coatings were evaluated by the R statistical software (The R Foundation for Statistical Computing, Vienna, Austria) using the SensoMineR package. Sensory data were processed by the principal component analysis (PCA) method.

Color parameters were statistically evaluated using the Unistat Tukey-HSD test procedure (Unistat Ltd., London, UK). The K samples test (Kruskal–Wallis test with Dunn multiple comparisons) by statistical software XLSTAT 2021 (Addinsoft, Paris, FR) was used for statistical comparison of the total area and nearest fissure distance.

## 3. Results and Discussion

### 3.1. Results of Sensory Analysis of Edible Packaging

#### 3.1.1. Quantitative Descriptive Analysis

**Figure 1:**
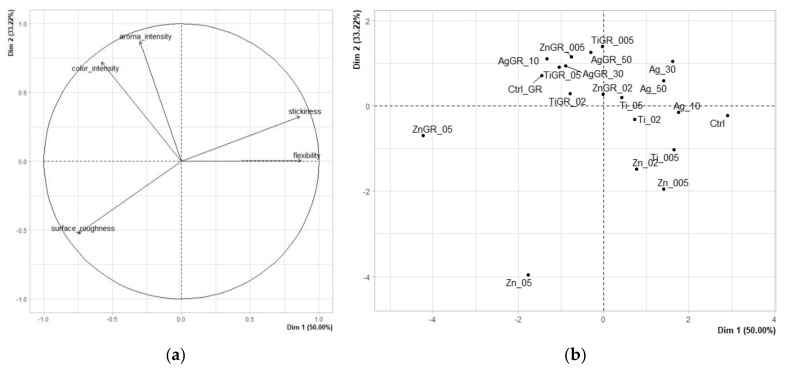
The PCA results of quantitative descriptive analysis of edible coatings: (**a**) Variables factor map. (**b**) Score plot for the mean points.

Factor analysis (Figure 1a) confirmed a close correlation between color intensity and odor intensity. Conversely, a negative correlation was confirmed between the descriptors of surface roughness (values ranging from the lowest for a smooth surface to the highest for a rough surface) and stickiness. The principal component analysis results graph explains 83.22% variability using the two principal components, with the first component explaining 50.00% and the second component 33.22% variability. From the sample map (Figure 1b) and from Table 2 showing statistically significant (*p* < 0.05) differences between individual edible films, it can be seen that there were a number of statistically significant differences between individual edible packaging samples. The remote sample Zn_05, characterized by high surface roughness and at the same time very low values of color intensity, odor intensity, stickiness, and flexibility, was clearly singled out on the sample map. The second remote sample was the ZnGR_05 sample, which was also characterized by high surface roughness, low stickiness, and flexibility, but at the same time statistically significantly higher odor and color intensity. Samples containing grape extract generally belonged to samples characterized by more intense color and aroma, due to the presence of typical anthocyanin pigments and a number of characteristic volatile aromatic substances [22,23]. The control sample without nanometals and also without grape extract, which was characterized by a smooth surface, low color and odor intensity, higher stickiness, and flexibility, also differed significantly. As reported by Marvizadeh et al. [24], increasing the proportion of zinc and titanium oxide nanoparticles caused a decrease in the flexibility of edible packaging, however, the packaging showed a smooth surface without pores or cracks. For the samples analyzed in this study, the flexibility values of the samples containing nanometals, but without the added grape extract, showed rather higher flexibility compared to the control sample. Surface roughness ranged from 1.89 to 8.49. As reported by Marvizadeh et al. [24], the addition of nanometals also contributed to a change in the instrumentally-determined color in the CIE*Lab* system by a significant reduction in values of *L* and at the same time increase in values of a* and b*, which showed a slight reddish and yellowish tinge. In our study, the color intensity of the packages was sensory evaluated, which was in most samples without grape extract also slightly higher compared to the control sample.

#### 3.1.2. Hedonic Analysis

A total of 11 samples, that were already evaluated by the panel in the previous step and reached the overall average score higher than 5 and were therefore evaluated as sensory acceptable, were afterward analyzed hedonically by untrained evaluators. 

**Figure 2:**
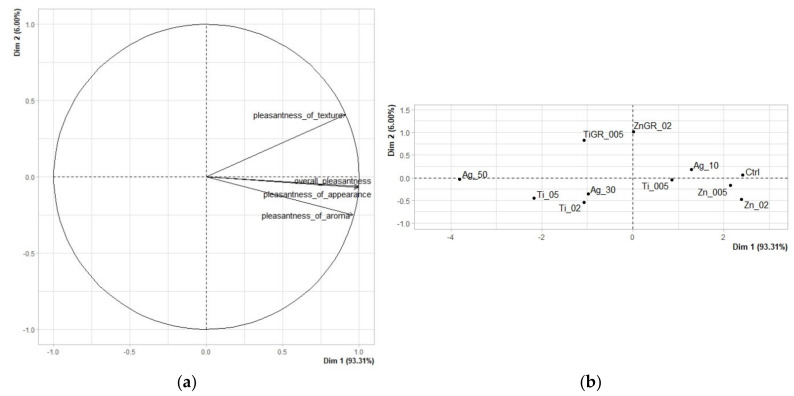
The PCA results of hedonic analysis of edible coatings: (**a**) Variables factor map. (**b**) Score plot for the mean points.

Factor analysis (Figure 2a) confirmed a close correlation between overall evaluation and appearance pleasantness, and to a lesser extent also odor pleasantness. The principal component analysis results graph explains 99.31% variability using the two principal components, with the first component explaining 93.31% and the second component only 6.00% variability. From the sample map (Figure 2b) and from Table 3 showing statistically significant (*p* < 0.05) differences between individual edible films, it can be seen that there were a number of statistically significant differences between individual edible packaging samples.

The remote sample Ag_50, characterized by the lowest values of pleasantness in all evaluated descriptors, was clearly singled out on the sample map. The second remote and worst-rated sample was Ti_05. The best evaluated was the control sample without nanometals and grape extract, which, however, was closely followed by samples with ZnO nanoparticles in concentrations of 0.2 and 0.05%. All these samples achieved low values in color and odor intensity and were characterized by a smooth surface with high flexibility. Thus, the high scores in the overall rating were related to their neutral nature, which is preferred for edible packaging [25,26]. Thus, samples without grape extract generally reached higher values, however, the ZnGR_02 sample achieved average values and was evaluated as generally acceptable. 

The addition of nanometals is important from a functional point of view and enables the production of packaging with antimicrobial activity [27]. However, samples with a higher concentration of nanometal addition generally achieved lower pleasantness.

#### 3.1.3. Assessment of the Probability of Purchasing Food Commodities in Edible Packaging

The results of the analysis showing how probable it is that different groups of food commodities packed in individual edible coatings will be purchased are shown in Table 4. As most of the values were below 3, it is clear that the evaluators could not imagine the use of the analyzed edible packaging for commodity groups such as meat products, milk products (cheeses), bakery products, or fruit and vegetables. The highest values, i.e., the highest probability of food purchase, were achieved by the control sample not containing grape extract or nanometals. Of the samples containing nanometals, the use of samples with 0.05% and 0.2% concentration of ZnO nanoparticles was the most conceivable for the evaluators for all evaluated types of commodities, and the Ti_005 sample for the packaging of fruit, vegetables, and milk products.

### 3.2. Evaluation of Color and Color Properties of Packaging

**Figure 3:**
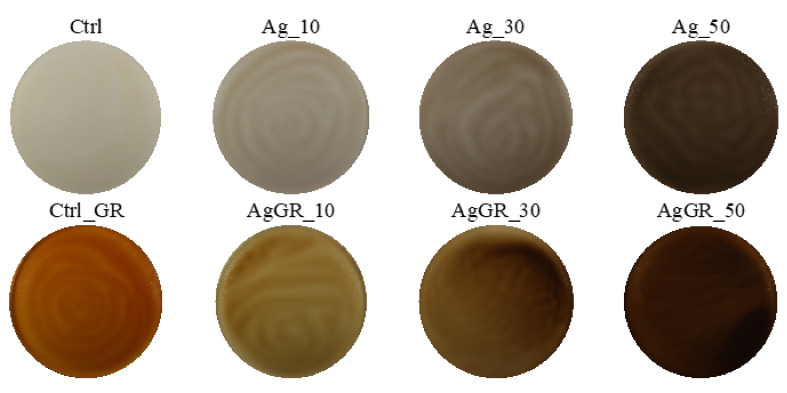
Digital images of edible coatings used for color analysis—samples with the addition of Ag nanoparticles.

**Figure 4:**
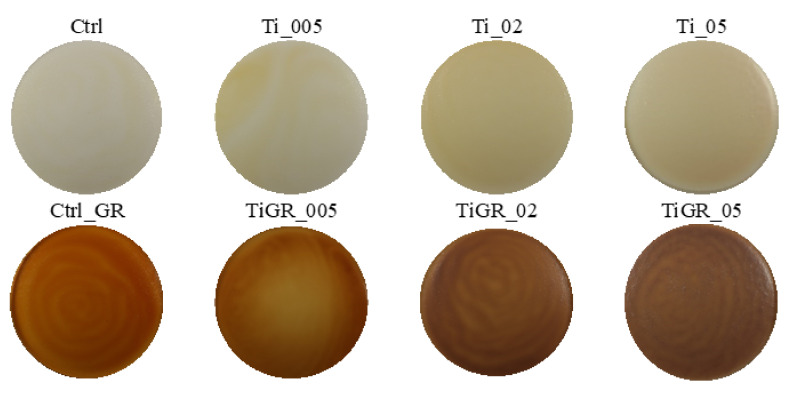
Digital images of edible coatings used for color analysis—samples with added TiO_2_ nanoparticles.

**Figure 5:**
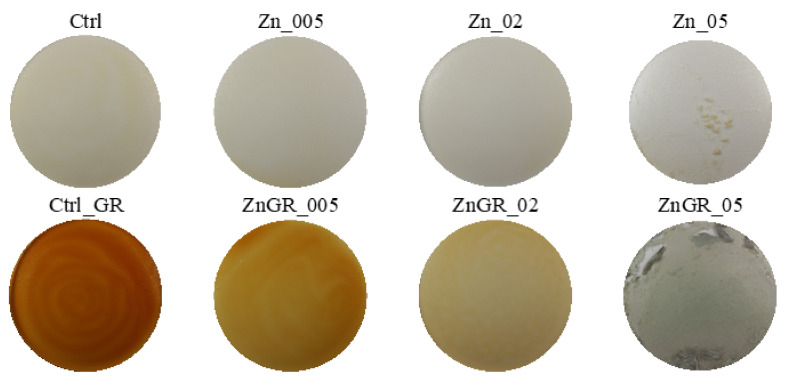
Digital images of edible coatings used for color analysis—samples with the addition of ZnO nanoparticles.

Digital images of individual samples of edible packaging with the addition of Ag, TiO_2,_ ZnO nanoparticles, and red grape extract are shown in Figure 3, Figure 4 and Figure 5. The results of the evaluation of individual color properties of packaging are summarized in Table 5, no statistically significant difference was found for samples with the same letter.

The samples with the addition of silver showed a decrease in the parameter compared to the control samples (Ctrl and Ctrl_GR) L*, on the contrary, there was an increase for parameters a* and b* (depending on the concentration added). 

**Figure 6:**
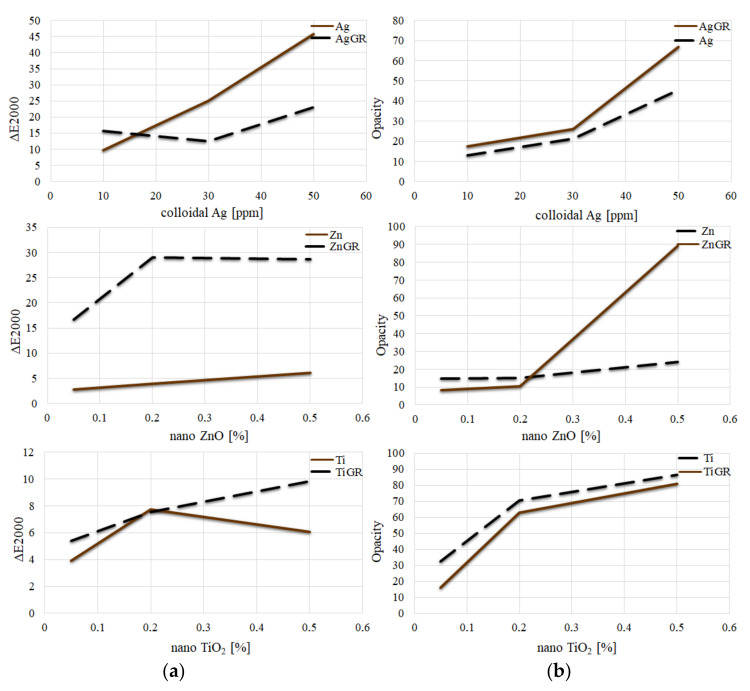
Evaluation of color properties of packaging: (**a**) A comparison of differences (∆E) of the samples in individual groups from the control sample Ctrl/Ctrl_GR. (**b**) Comparison of sample opacity with individual additions.

A comparison of the differences (∆E) of samples in individual groups from the control samples Ctrl and Ctrl_GR is shown in Figure 6a. A statistically significant difference (*p* < 0.05) was demonstrated when comparing the measurement results and the calculated ΔE parameter. As the concentration of Ag and ZnO nanoparticles addition was increased, the difference (ΔE) between the samples and the control sample without the addition of grape extract clearly increased as well. However, in the case of samples containing grape extract, this clear trend was only confirmed in the case of the addition of TiO_2_ nanoparticles.

In addition, the reduction in lightness (L) can be attributed to the opacity of the silver nanoparticles [11]. According to a study by Rhim et al. (2013) who monitored the effect of the addition of silver nanoparticles, the parameter ΔE increases, and the color of the packaging changes to dark brown depending on the concentration of silver nanoparticles [28]. We achieved similar results in our study.

For packaging samples with the addition of TiO_2_ nanoparticles, ΔE increased, which is in line with the results of a similar study by Dash et al. (2019) [29]. Coatings containing TiO_2_ should also have a higher parameter of L*, b*, and ΔE with increasing concentration [30]. Our study delivered corresponding values.

In their work, Wardana et al. (2018) reported that increasing the addition of ZnO nanoparticles increases ΔE, which corresponds to our results [31].

#### 3.2.1. Opacity

A comparison of differences in the transparency of packaging, which was calculated from the L value obtained from images against a white and a black background, is shown in Figure 6b.

The lowest opacity values were reached by the control samples (Ctrl and Ctrl_GR). All other samples had a higher value, which increased with increasing concentration. As a result, the samples were less transparent. The transparency of the packaging, therefore, decreased with increasing concentrations. This trend was clear both for samples with and without grape extract. Individual results are summarized in Table 5, no statistically significant difference was found for samples with the same letter.

#### 3.2.2. Whiteness Index; Yellowness Index

Samples containing 10% grape addition had a lower Whiteness Index than samples without the additive. In their study, Li et al. (2019) report that the reduction in whiteness is probably due to the formation of dark compounds formed during the Maillard reaction [20]. In the case of the addition of Ag nanoparticles, the Whiteness Index decreased with increasing concentrations, regardless of the addition of grape extract. The addition of ZnO and TiO_2_ nanoparticles increased the Whiteness Index values rather slightly, which is consistent with the results in a study by Dash et al. (2019) [29]. Coatings containing TiO_2_ should also have higher parameters of L*, b*, ΔE, as well as Whiteness Index, with increasing concentration [30]. We achieved similar results in our study.

**Figure 7:**
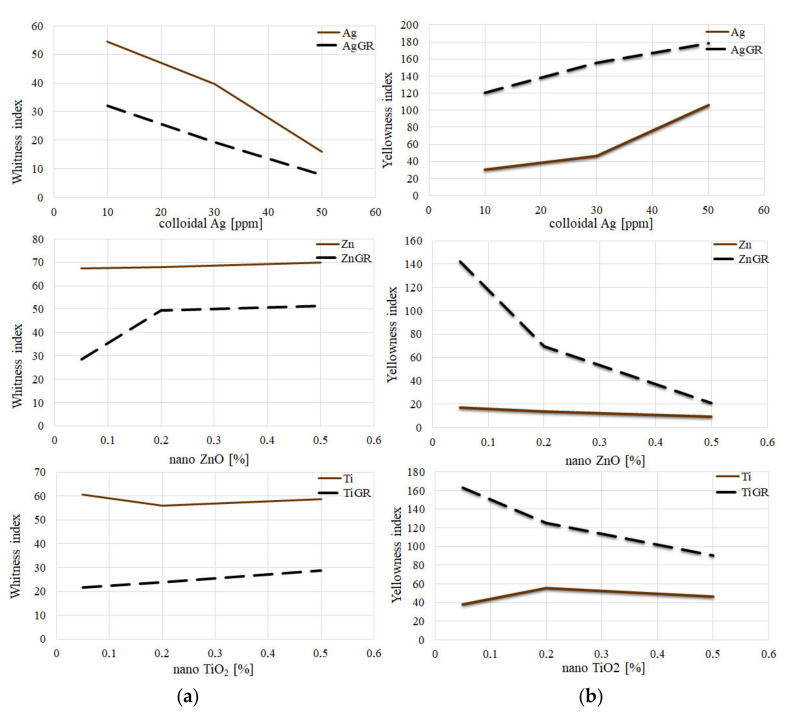
Evaluation of color properties of packaging: (**a**) Comparison of Whiteness Index with individual additions. (**b**) Comparison of Yellowness Index with individual additions.

Samples with the addition of grape extract generally had a higher Yellowness Index than samples without this additive. In the case of the addition of Ag nanoparticles, the Yellowness Index increased with increasing concentrations, regardless of the addition of grape extract. On the contrary, the addition of ZnO and TiO_2_ nanoparticles rather reduced the values of the Yellowness Index (Figure 7).

### 3.3. Evaluation of the Surface of Edible Packaging

**Figure 8:**
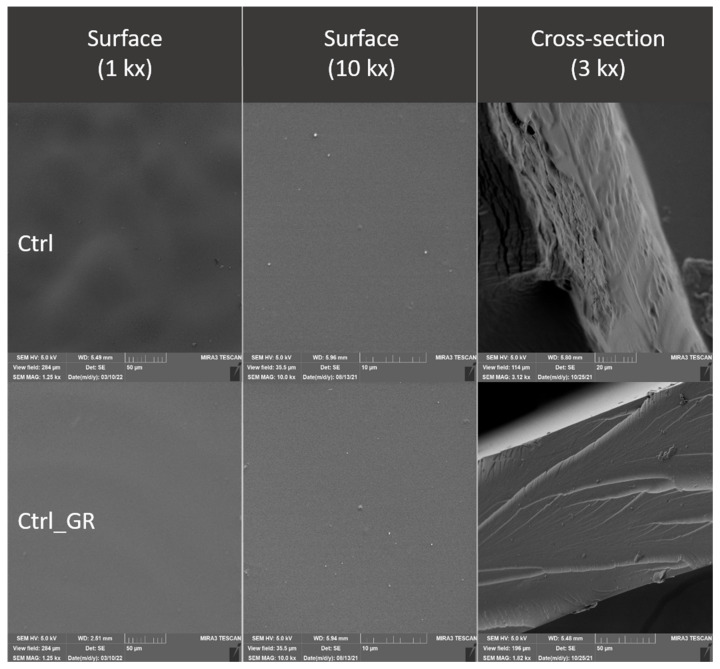
Surface and fracture microstructure of control samples of edible packaging.

The comparison of the Ctrl control and the Ctrl_GR containing grape extract shows a minimum difference. At Ctrl_GR, there is a small occurrence of insoluble particles of grape extracts confirmed both, on the surface as well as in the cracks (Figure 8 Ctrl_GR). The insoluble matter distribution is random and there is no separation of thermodynamic phases. The total number of cracks and the distance between the cracks were the lowest in the control samples, and statistical differences between the control and other samples were confirmed (*p* ˂ 0.05) (Table 6 and Table 7). The difference in comparison with the control containing the addition of the grape extract was not proved at the nearest object distance in ZnGr_005, the difference was not confirmed in the sample with the addition of grape extract in ZnGR_02.

**Figure 9:**
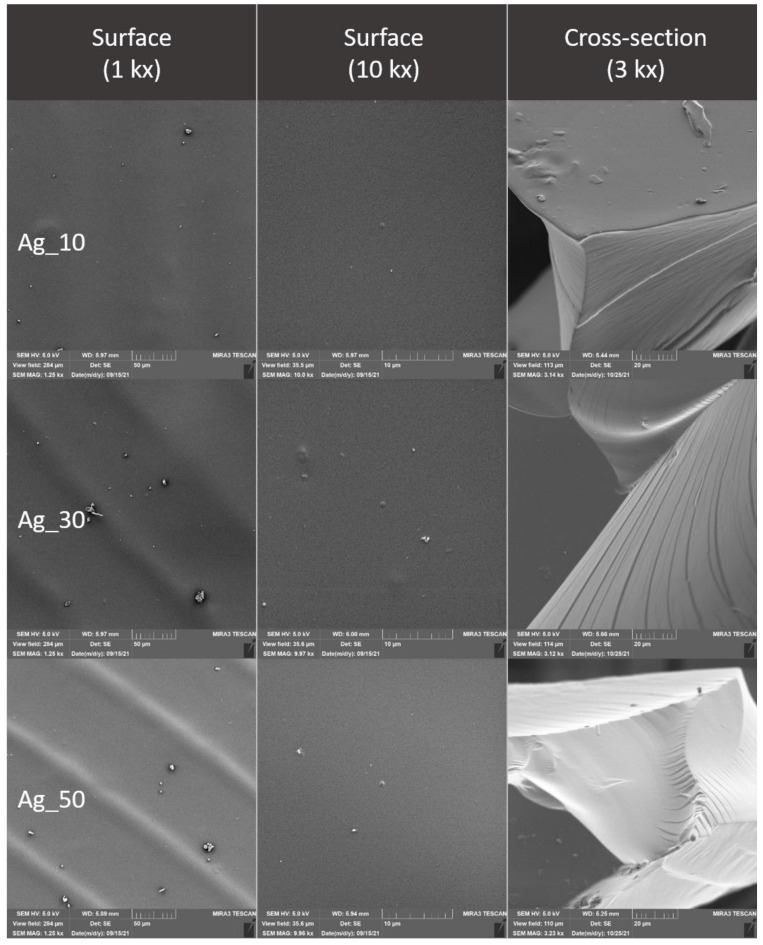
Surface and fracture microstructure of edible packaging with the addition of colloidal silver nanoparticles.

**Figure 10:**
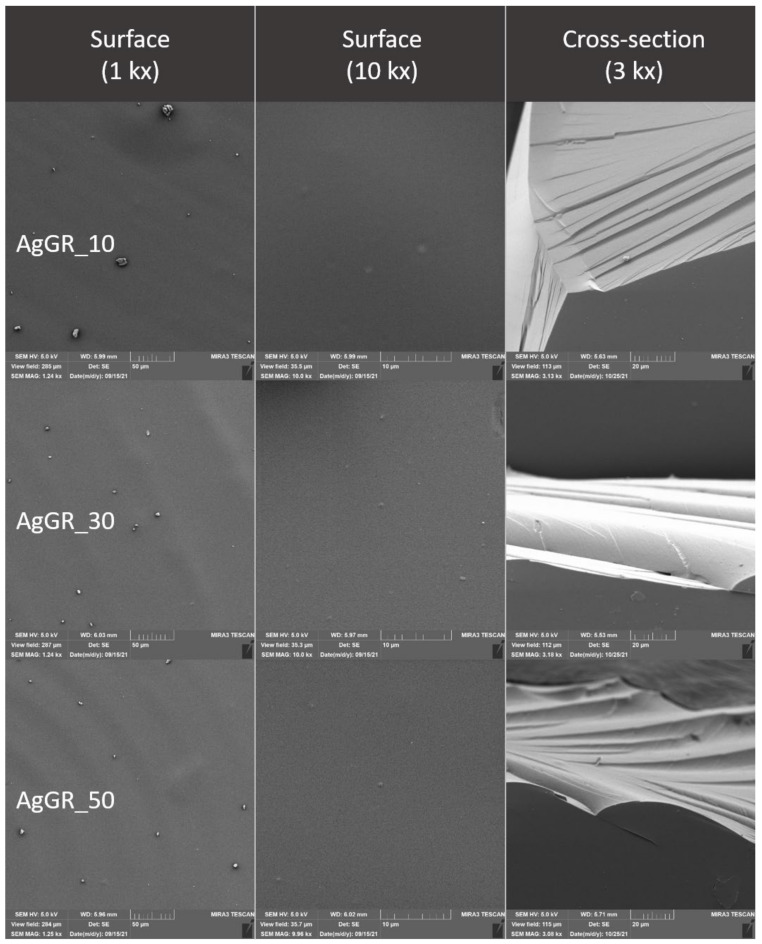
Surface and fracture microstructure of edible packaging with the addition of colloidal silver nanoparticles and grape extract.

As concluded by Bakhy et al. (2018), metal oxide nanoparticles, such as nano-Ag, improve the mechanical and barrier properties of biodegradable films [32]. Colloidal silver, like other nanoparticles, forms structures that roughen the surface or are visible at cracks. The silver distribution is uneven and strongly adheres to the chitosan gel [33]. Many authors describe the shape characteristics of nanosilver differently, when they even state the size of silver 80–110 nm, i.e., above the well-known term of nanoparticles (max 100 nm). In the case of our study, the presence of colloidal silver was not confirmed (Figure 9 and Figure 10). However, the addition affected the nearest object distance when there was a statistically significant decrease in the concentration of Ag30 and AgHr30 (*p* ˂ 0.05). For the area, this phenomenon was confirmed only for samples without added extract (Table 6 and Table 7).

**Figure 11:**
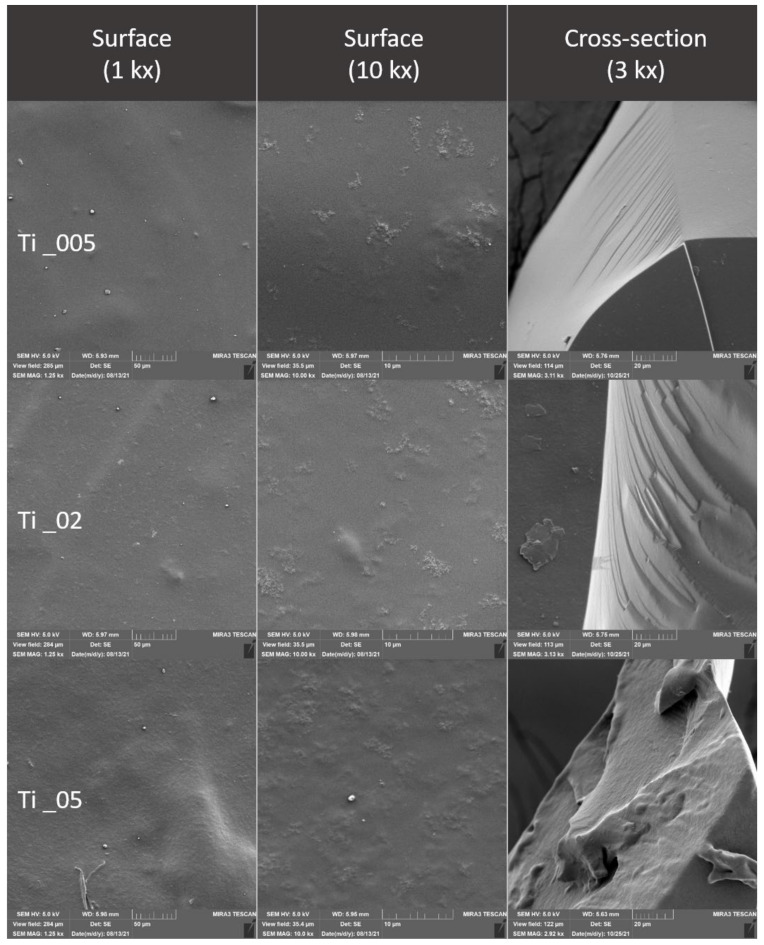
Surface and fracture microstructure of edible packaging with the addition of TiO_2_ nanoparticles.

**Figure 12:**
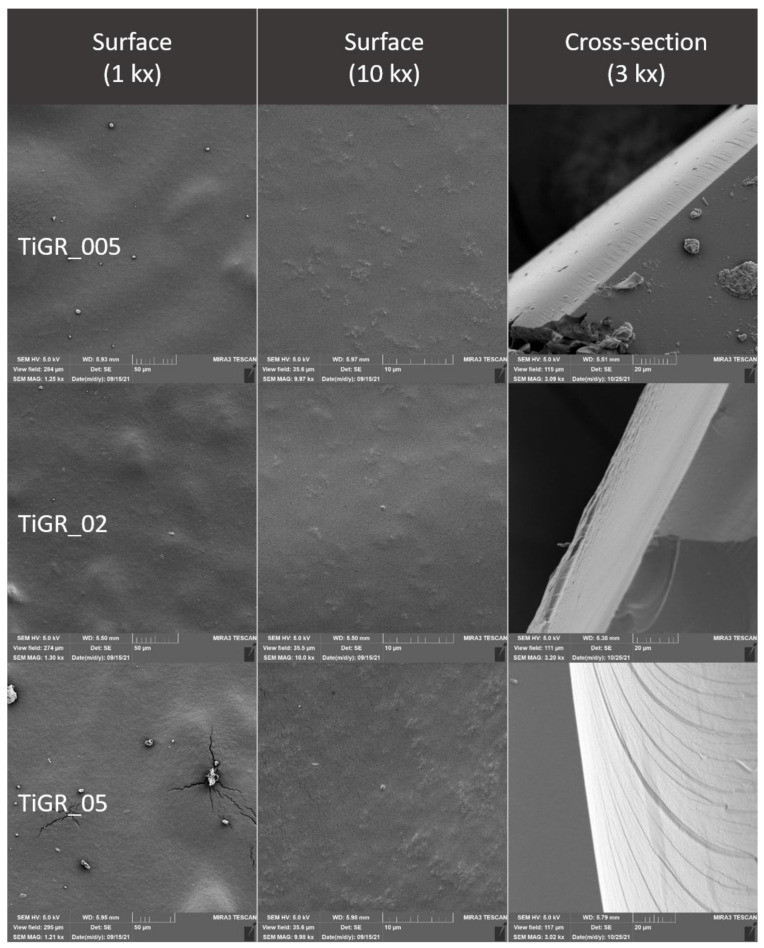
Surface and fracture microstructure of edible packaging with the addition of TiO_2_ nanoparticles and grape extract.

The microstructure of the surface of edible coatings was also observed after the addition of TiO_2_ (Figure 11 and Figure 12). As several authors claim, TiO_2_ forms polygonal and oval crystals with an average size of 1.84–40 µm [32]. Our results also confirmed the oval shape of TiO_2_ crystals. Crystal formation was observed on the surface, but also in the fractures of the packaging. This result is partly consistent with [34], where they confirmed the formation of crystals only in the fractures, while they were not observed on the surface. The difference may be due to the different concentrations, where a uniform concentration of 1% TiO_2_ was used in this study, however, the concentrations in our study were lower, namely 0.05–0.5%. Many authors confirm the properties we observed, when crystal formation occurs in the entire range of biodegradable films [35]. From the fissure area comparison, no statistical difference was confirmed for samples from Ti_005 and 02 concentrations (*p* > 0.05), the concentration with the highest Ti_05 addition was statistically different (*p* < 0.05) (Table 7). The nearest object distance was different between samples in all cases (*p* < 0.05) (Table 6).

**Figure 13:**
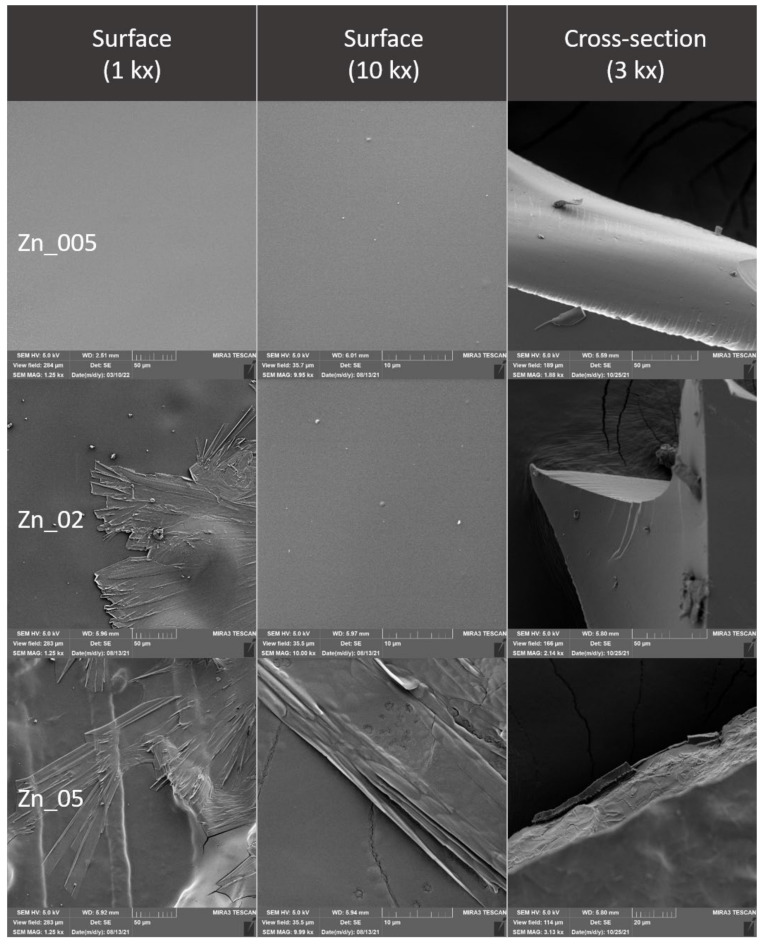
Surface and fracture microstructure of edible packaging with the addition of ZnO nanoparticles.

**Figure 14:**
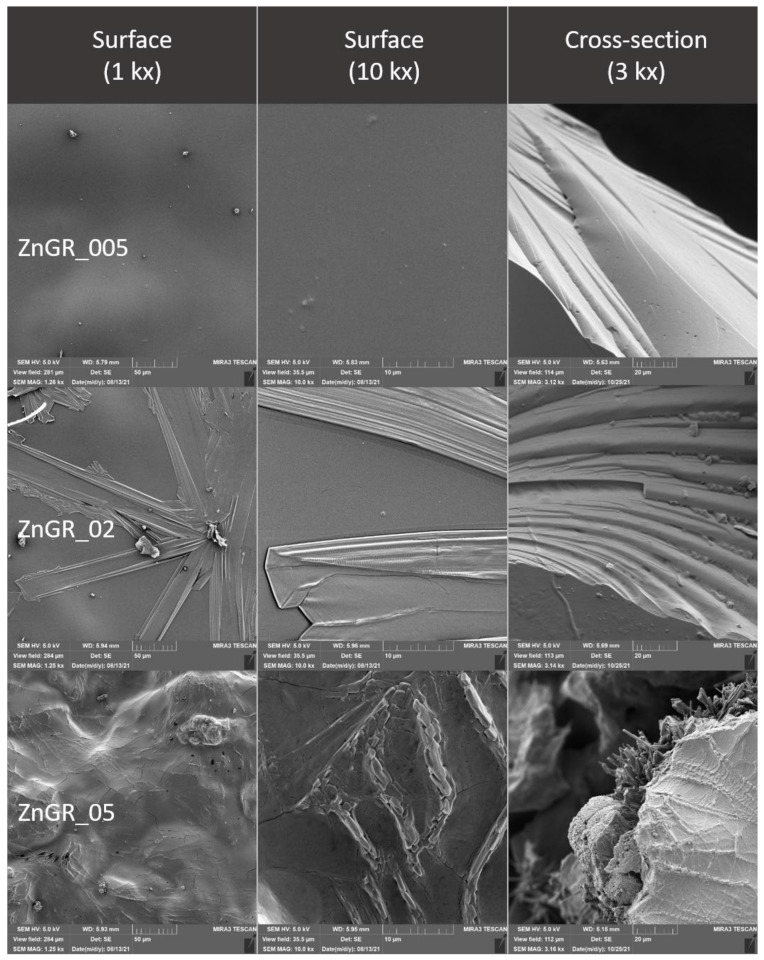
Surface and fracture microstructure of edible packaging with the addition of ZnO nanoparticles and grape extract.

**Figure 15:**
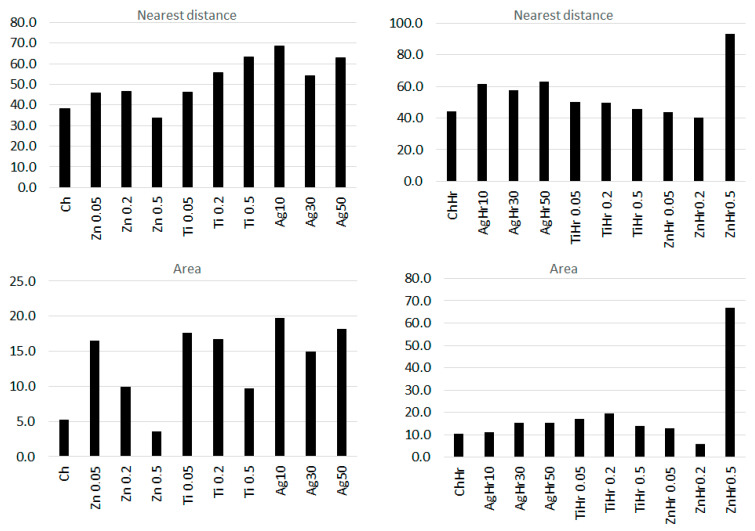
Comparison of the nearest object distance and area in edible films.

The addition of ZnO to edible package resulted in a roughening of the surface of the package in comparison with the control (Figure 9, Figure 13, and Figure 14). From the point of view of the microstructure, ZnO particles have a regular circular structure [5] which was also reflected in the structure of the formed edible packaging. Similar findings are also confirmed by other studies, where ZnO also formed an irregular structure visible in SEM, from the addition of 0.1–1.0% upwards [5]. At a higher magnification, the circular structure of ZnO projected on the surface was visible. In some cases, other structures may be formed, such as crystalline efflorescence [20]. Crystalline efflorescence was confirmed in this study, especially on the surface of the packaging. Crystal formation was accompanied by phase segregation, which was caused by thermodynamic incompatibilities commonly described in biofilms [36]. In the case of Zn and ZnGR samples at concentrations of 0.2–0.5%, cracks breaking the surface of the edible coating were also observed. The origin of the cracks was due to the different thermodynamic properties of the two materials. Crystals and phase segregations were observed in the ZnGR 0.05 and 0.2% samples at the fracture. The ZnGR_05 sample was characterized by large uneven crystals along with peeling parts due to phase segregation. The particle distribution was uneven on the surface as well as in the fracture of the package, which is in agreement with the results of other studies [5,37]. Uneven distribution was also confirmed by the largest nearest object distance and area in samples with the addition of grape extract at the highest concentration of ZnHr05. ZnHr05 differed statistically from lower concentrations (*p* ˂ 0.05) (Table 6 and Table 7, Figure 15). On the other hand, in samples without the addition of grape extract, the opposite connection was demonstrated in the area where ZnHr_05 was the lowest. Nevertheless, at the nearest object distance, Zn_02 was the lowest. These results indicate the randomness of crack formation within the edible films (Table 6 and Table 7, Figure 13, Figure 14 and Figure 15). 

Differences between samples with 10% grape extract and samples without the extract were confirmed in all samples examined. The presence of unspecified structures originating from the extraction process was confirmed in all samples of edible packaging containing the addition of grape extract. However, their incorporation into the chitosan network was confirmed, their presence caused a biofilm prominence, which is caused by the adherence of chitosan to plant tissues.

## 4. Conclusions

The addition of grape extract generally reduced the flexibility of the samples and at the same time increased the intensity of odor and color to the extent that only two samples containing this extract and containing nanometals at the same time achieved a satisfactory score in the overall evaluation. There was no clear trend in terms of the effect of different nanometallic concentration additions on the quantitatively descriptively evaluated descriptors. However, the hedonic evaluation revealed that the addition of nanometals and their increasing concentration generally caused a deterioration in sample acceptability. In spite of that, the overall evaluation was higher than 5 in 9 out of the total number of 18 samples containing nanometals. From a sensory point of view, the addition of ZnO nanoparticles in concentrations of 0.05 and 0.2% appeared to be the most favorable of all nanometals.

The addition of nanometals also caused statistically significant changes in L*, a*, and b* values. The sample transparency generally decreased with increasing concentration of nanoparticle addition, regardless of the type of nanoparticle. The addition of grape extract significantly affected the samples in the Whiteness Index and Yellowness Index parameters, while the grape addition decreased the Whiteness Index values and increased the Yellowness Index values.

Electron microscopy also confirmed the differences between the control and the individual samples caused by the addition of nanometals, both on the basis of the evaluation of SEM images and on the basis of the evaluation of cracks in the formed edible film. The difference was not confirmed only between the control and the lowest concentrations for ZnGR_005 for the nearest object distance and ZnGR_02 for the area. The differences were confirmed on the surface and also in the fracture of edible chitosan-based packaging. Differences between samples with and without the addition of grape extract were also confirmed. In addition, our results confirmed that packaging handling causes structural changes [15]. Therefore, for evaluation purposes, we recommend direct gel formation on a conductive target.

## Figures and Tables

**Table 1:** Composition of the coatings produced.

Sample	Composition
Ctrl	Chitosan + 1% lactic acid + glycerol
CtrlGR	Chitosan + 1% lactic acid + 10% extract * + glycerol
Zn_005	Chitosan + 1% lactic acid + 0.05% nano ZnO + glycerol
Zn_02	Chitosan + 1% lactic acid + 0.2% nano ZnO + glycerol
Zn_05	Chitosan + 1% lactic acid + 0.5% nano ZnO + glycerol
Ti_005	Chitosan + 1% lactic acid + 0.05% nano TiO_2_ + glycerol
Ti_02	Chitosan + 1% lactic acid + 0.2% nano TiO_2_ + glycerol
Ti_05	Chitosan + 1% lactic acid + 0.5% nano TiO_2_ + glycerol
Ag_10	Chitosan + 1% lactic acid + colloidal Ag 10 ppm + glycerol
Ag_30	Chitosan + 1% lactic acid + colloidal Ag 30 ppm + glycerol
Ag_50	Chitosan + 1% lactic acid + colloidal Ag 50 ppm + glycerol
ZnGR_005	Chitosan + 1% lactic acid + 0.05% nano ZnO + 10% extract * + glycerol
ZnGR_02	Chitosan + 1% lactic acid + 0.2% nano ZnO + 10% extract * + glycerol
ZnGR_05	Chitosan + 1% lactic acid + 0.5% nano ZnO + 10% extract * + glycerol
TiGR_005	Chitosan + 1% lactic acid + 0.05% nano TiO_2_ + 10% extract * + glycerol
TiGR_02	Chitosan + 1% lactic acid + 0.2% nano TiO_2_ + 10% extract * + glycerol
TiGR_05	Chitosan + 1% lactic acid + 0.5% nano TiO_2_ + 10% extract * + glycerol
AgGR_10	Chitosan + 1% lactic acid + colloidal Ag 10 ppm + 10% extract * + glycerol
AgGR_30	Chitosan + 1% lactic acid + colloidal Ag 30 ppm + 10% extract * + glycerol
AgGR_50	Chitosan + 1% lactic acid + colloidal Ag 50 ppm + 10% extract * + glycerol

* Red grape extract.

**Table 2:** Adjusted mean values of evaluation within the quantitative descriptive analysis of edible packaging.

	Flexibility	Stickiness	Aroma Intensity	Color Intensity	Surface Roughness
**Ctrl**	8.041	4.625	3.636	2.618	2.986
**Ag_10**	8.005	3.630	4.216	3.158	2.592
**Ti_005**	7.605	3.830	3.416	3.658	3.592
**Ag_30**	8.205	4.330	5.216	4.458	3.092
**Zn_005**	6.705	3.330	3.016	1.858	3.592
**Ag_50**	8.705	4.330	4.616	5.858	4.092
**Zn_02**	7.705	2.830	3.916	2.058	4.492
**Ti_02**	7.905	3.13	3.716	6.358	3.492
**Ti_05**	7.605	3.13	4.116	6.758	3.592
**ZnGR_02**	7.705	2.73	4.616	6.558	3.992
**TiGR_005**	4.405	3.43	4.616	7.958	1.892
**AgGR_50**	5.205	3.33	4.816	8.958	3.192
**ZnGR_005**	4.905	3.13	4.916	7.658	3.492
**TiGR_02**	6.605	2.93	4.416	8.558	5.192
**AgGR_30**	4.305	3.33	4.616	8.658	3.892
**TiGR_05**	6.305	2.73	5.116	8.558	4.792
**Zn_05**	4.005	2.03	2.516	2.858	8.392
**CtrlGR**	3.405	3.13	4.816	7.758	4.292
**AgGR_10**	4.605	3.23	5.416	8.058	4.692
**ZnGR_05**	1.705	1.63	5.316	7.358	8.492

Values highlighted with green color are statistically significantly higher, values highlighted with orange color are statistically significantly lower (*p* < 0.05).

**Table 3:** Adjusted mean scores of hedonic evaluation of edible coatings.

	Texture Pleasantness	Aroma Pleasantness	Appearance Pleasantness	Overall Pleasantness
**Ag_50**	5.800	4.708	3.031	4.569
**Ti_05**	6.000	5.123	4.185	5.262
**TiGR_005**	6.677	5.031	4.785	5.708
**Ti_02**	6.215	5.385	4.815	5.723
**Ag_30**	6.308	5.369	4.785	5.769
**ZnGR_02**	7.031	5.308	5.215	6.000
**Ti_005**	6.754	5.538	6.262	6.723
**Ag_10**	6.954	5.631	6.415	6.769
**Zn_005**	6.985	5.815	7.138	7.262
**Zn_02**	6.938	5.954	7.323	7.323
**Ctrl**	7.169	5.908	7.031	7.246

Values highlighted with green color are statistically significantly higher, values highlighted with orange color are statistically significantly lower (*p* > 0.05).

**Table 4:** Adjusted mean scores of the probability of purchasing a certain commodity in an edible coating.

	Meat Products	Milk Products (Cheese)	Bakery Products	Fruit	Vegetables
**Ag_50**	1.865	1.676	1.703	1.595	1.595
**ZnGR_02**	3.081	2.027	2.270	2.189	2.162
**Ti_05**	2.351	3.351	2.081	2.162	2.162
**TiGR_005**	3.459	1.946	2.162	2.243	2.081
**Ag_30**	2.676	2.324	2.514	2.432	2.405
**Ti_02**	2.270	3.378	2.351	2.514	2.459
**Ag_10**	2.946	2.784	2.649	2.919	3.000
**Ti_005**	2.973	3.405	2.973	3.216	3.270
**Ctrl**	3.000	3.405	2.649	3.676	3.459
**Zn_02**	3.703	3.595	3.378	4.000	3.838
**Zn_005**	3.730	3.811	3.432	4.324	4.297

Values highlighted with green color are statistically significantly higher, values highlighted with orange color are statistically significantly lower (*p* > 0.05).

**Table 5:** Results of evaluation of color properties of packaging.

	L*	a*	b*	ΔE	Opacity	Whiteness Index	Yellowness Index
**Ag_10**	56.15 ± 0.21	0.56 ± 0.04	11.90 ± 0.08	9.63 ± 0.31 ^l^	17.57 ± 0.37	54.56 ± 0.20	30.28 ± 0.21
**Ag_30**	41.36 ± 0.18	2.66 ± 0.02	13.44 ± 0.09	25.11 ± 0.36	26.11 ± 0.47	39.78 ± 0.16	46.43 ± 0.16 ^v^
**Ag_50**	17.19 ± 0.24	5.43 ± 0.05 ^e^	12.76 ± 0.09	45.88 ± 0.41	66.86 ± 1.67	16.04 ± 0.22 ^t^	106.09 ± 0.87
**AgGR_10**	42.26 ± 0.28	5.33 ± 0.12 ^e^	35.58 ± 0.15	15.57 ± 0.17	12.95 ± 0.13 ^o^	31.97 ± 0.17	120.29 ± 0.45
**AgGR_30**	24.11 ± 0.45	10.32 ± 0.24 ^f^	26.30 ± 0.16	12.43 ± 0.23	21.23 ± 1.03	19.02 ± 0.42	155.93 ± 2.38
**AgGR_50**	9.06 ± 0.20	10.45 ± 0.14 ^f^	11.32 ± 0.30 ^g^	23.04 ± 0.40	45.35 ± 1.27	7.76 ± 0.14	178.45 ± 0.99
**Ti_005**	64.66 ± 0.25 ^a^	−2.20 ± 0.12 ^d^	17.12 ± 0.24	3.91 ± 0.13 ^i^	32.42 ± 0.32	60.67 ± 0.26	37.82 ± 0.56
**Ti_02**	63.41 ± 0.28	−1.76 ± 0.05	24.51 ± 0.09	7.75 ± 0.18 ^k^	70.54 ± 0.49	55.92 ± 0.26	55.23 ± 0.40
**Ti_05**	64.27 ± 0.36 ^a^	−0.51 ± 0.02	20.96 ± 0.09 ^h^	6.05 ± 0.20 ^j^	86.48 ± 0.85	58.57 ± 0.35	46.60 ± 0.44 ^v^
**TiGR_005**	35.16 ± 0.30	17.93 ± 0.08	40.17 ± 0.23	5.37 ± 0.07	16.03 ± 0.35 ^s^	21.64 ± 0.13 ^u^	163.22 ± 0.47
**TiGR_02**	30.62 ± 0.26	16.42 ± 0.05	26.91 ± 0.11	7.54 ± 0.27 ^k^	62.91 ± 1.01	23.80 ± 0.20	125.55 ± 0.59
**TiGR_05**	33.41 ± 0.31 ^b^	13.46 ± 0.07	21.08 ± 0.13 ^h^	9.85 ± 0.11 ^l^	81.05 ± 0.96	28.87 ± 0.26 ^u^	90.14 ± 0.60
**Zn_005**	68.42 ± 0.36	−1.07 ± 0.05	8.03 ± 0.05	2.75 ± 0.25	14.65 ± 0.22 ^qr^	67.39 ± 0.34	16.78 ± 0.16
**Zn_02**	68.66 ± 0.39 ^c^	−0.82 ± 0.08	6.52 ± 0.09	3.88 ± 0.17 ^i^	15.01 ± 0.29 ^prs^	67.98 ± 0.40	13.56 ± 0.25
**Zn_05**	70.33 ± 0.42 ^c^	−0.28 ± 0.03	4.38 ± 0.10	6.05 ± 0.22 ^j^	24.06 ± 0.76	70.01 ± 0.42	8.90 ± 0.23
**ZnGR_005**	47.00 ± 0.35	10.09 ± 0.15	46.77 ± 0.20	16.70 ± 0.45	8.38 ± 0.18 ^n^	28.60 ± 0.20	142.17 ± 0.70
**ZnGR_02**	57.89 ± 0.42	0.16 ± 0.10	28.16 ± 0.09	29.09 ± 0.49 ^m^	10.45 ± 0.20	49.34 ± 0.32	69.50 ± 0.42
**ZnGR_05**	52.11 ± 0.35	−2.08 ± 0.04 ^d^	7.70 ± 0.06	28.67 ± 0.29 ^m^	89.04 ± 0.82	51.45 ± 0.35	21.11 ± 0.25
**Ctrl**	66.91 ± 0.37	−1.41 ± 0.03	11.54 ± 0.10 ^g^	-	14.13 ± 0.55 ^opq^	64.93 ± 0.40	24.63 ± 0.32
**Ctrl_GR**	33.89 ± 0.43 ^a^	27.49 ± 0.18	43.91 ± 0.34	-	7.59 ± 0.20 ^n^	16.01 ± 0.16 ^t^	185.12 ± 0.96

* Samples marked with indices within the column do not differ statistically significantly from each other.

**Table 6:** Nearest object distance between fissures.

Sample	Nearest Object Distance (nm)	Sample	Nearest Object Distance (nm)
**Ctrl**	38.25 ± 23.12 ^a^	**Ctrl**	43.95 ± 23.77 ^a^
**Ag_10**	68.49 ± 25.32 ^b^	**AgGR_10**	61.42 ± 23.95 ^b^
**Ag_30**	54.27 ± 24.63 ^c^	**AgGR_30**	57.56 ± 24.26 ^c^
**Ag_50**	62.95 ± 24.77 ^d^	**AgGR_50**	62.86 ± 23.42 ^d^
**Ti_005**	46.11 ± 19.93 ^e^	**TiGR_005**	50.18 ± 23.86 ^e^
**Ti_02**	55.7 ± 24.56 ^f^	**TiGR_02**	49.46 ± 23.84 ^f^
**Ti_05**	63.26 ± 25.09 ^g^	**TiGR_05**	45.84 ± 23.76 ^g^
**Zn_005**	46.49 ± 24.07 ^h^	**ZnGR_005**	43.48 ± 23.2 ^a^
**Zn_02**	33.54 ± 27.62 ^e^	**ZnGR_02**	40.36 ± 21.63 ^h^
**Zn_05**	43.48 ± 23.2 ^i^	**ZnGR_05**	93.06 ± 31.52 ^i^

**Table 7:** Total fissure area.

Sample	Area (µm^2^)	Sample	Area (µm^2^)
**Ctrl**	0.52 ± 1.53 ^a^	**Ctrl**	1.04 ± 3.72 ^a^
**Ag_10**	1.97 ± 3.47 ^b^	**AgGR_10**	1.12 ± 1.65 ^b^
**Ag_30**	1.5 ± 2.89 ^c^	**AgGR_30**	1.55 ± 3.08 ^c^
**Ag_50**	1.82 ± 3.87 ^d^	**AgGR_50**	1.54 ± 2.94 ^d^
**Ti_005**	1.77 ± 14.22 ^e^	**TiGR_005**	1.71 ± 4.13 ^e^
**Ti_02**	1.67 ± 3.95 ^e^	**TiGR_02**	1.97 ± 5.78 ^e^
**Ti_05**	0.97 ± 1.47 ^f^	**TiGR_05**	1.41 ± 4 ^f^
**Zn_005**	1.66 ± 6.02 ^g^	**ZnGR_005**	1.31 ± 3.69 ^g^
**Zn_02**	0.99 ± 1.93 ^g^	**ZnGR_02**	0.6 ± 6 ^a^
**Zn_05**	0.35 ± 3.65 ^h^	**ZnGR_05**	6.67 ± 6.59 ^h^

## Data Availability

Not applicable.
